# Esophageal cancer presenting with atrial fibrillation: A case report

**DOI:** 10.1186/1752-1947-2-292

**Published:** 2008-09-08

**Authors:** Ulas Darda Bayraktar, Alix Dufresne, Soley Bayraktar, Roland Royston Purcell, Ofem Ibiah Ajah

**Affiliations:** 1 Division of Hematology and Oncology, Sylvester Comprehensive Cancer Center, 1475 NW 12th Ave, St# 3400 (D8-4), Miami, FL 33136, USA; 2 Division of Cardiology, Interfaith Medical Center, 1545 Atlantic Ave, Brooklyn, NY 11213, USA; 3 Department of Surgery, Interfaith Medical Center, 1545 Atlantic Ave, Brooklyn, NY 11213, USA; 4 Division of Gastroenterology, Interfaith Medical Center, 1545 Atlantic Ave, Brooklyn, NY 11213, USA

## Abstract

**Introduction:**

Atrial fibrillation was previously reported in patients with esophageal cancer as a complication of total esophagectomy or photodynamic therapy. Here, we propose that atrial fibrillation may also be caused by external compression of the left atrium by esophageal cancer.

**Case presentation:**

We present a 58-year-old man who developed atrial fibrillation with rapid ventricular rate in the emergency room while being evaluated for dysphagia and weight loss. Atrial fibrillation lasted less than 12 hours and did not recur. Echocardiogram did not reveal any structural heart disease. A 10-cm, ulcerated mid-esophageal mass was seen during esophagogastroscopy. Microscopic examination showed squamous cell carcinoma. Computed tomography of the chest revealed esophageal thickening compressing the left atrium.

**Conclusion:**

External compression of the left atrium was previously reported to provoke atrial fibrillation. Similarly, esophageal cancer may precipitate atrial fibrillation by mechanical compression of the left atrium or pulmonary veins, triggering ectopic beats in susceptible patients.

## Introduction

Atrial fibrillation (AF) is a common arrhythmia and its prevalence increases with age. It is usually associated with underlying heart disease, of almost any cause, complicated by heart failure and atrial enlargement. Most common underlying disorders are hypertensive heart disease, coronary artery disease, valvular heart disease, hyperthyroidism, and alcoholism. The majority of AF episodes were found to be triggered by atrial ectopic beats from muscle fibers extending from the left atrium into the pulmonary veins [1]. Hence, radiofrequency catheter ablation of the pulmonary veins is effective for curing atrial fibrillation in selected cases, which may be complicated with atrioesophageal fistulas due to the proximity of the esophagus to the left atrium [2].

Esophageal cancer (EC) is a relatively rare malignancy in the United States with a poor prognosis. The majority of ECs are squamous cell carcinoma (SCC) and adenocarcinoma (AC). Dysphagia and weight loss are the two most common presenting symptoms. The majority of SCCs are located in the midportion of the esophagus where it is closely related to the posterior wall of the left atrium.

AF was previously reported in patients with EC as a complication of total esophagectomy or photodynamic therapy [3,4]. This may be due to manipulation of the left atrium during the surgical procedure or deep penetration of light waves affecting the left atrium during the photodynamic therapy. Here, we will present a rare case, a patient with AF who was diagnosed with EC compressing the left atrium.

## Case presentation

A 58-year-old black man from the Caribbean was referred by his primary care physician for evaluation of dysphagia and weight loss. He reported a 2-month history of progressively worsening dysphagia with solids only and weight loss of 10 kg over a period of 2 months. He denied cough, regurgitation, hoarseness, palpitations, and dyspnea. Past medical history was significant for hypertension (HTN) for 5 years which had been treated with valsartan and hydrochlorothiazide. He denied any history of cardiovascular problems or arrhythmias. He quit smoking 7 years ago and denied drinking alcohol. There was no other significant medical, family or social history.

Initial physical examination revealed regular heart rhythm with a rate of 81/minute. Abdominal and chest examinations were normal. Initial electrocardiogram (EKG) in the emergency room (ER) showed normal sinus rhythm with a rate of 68/minute and left ventricular hypertrophy (LVH). Chest X-ray revealed multiple nodules in both lung fields without cardiomegaly. Laboratory tests revealed mild normochromic, normocytic anemia with hemoglobin of 12.9 g/dl. Biochemical and coagulation studies were within normal limits. His serum potassium level was 4.2 mEq/liter.

**Figure 1:**
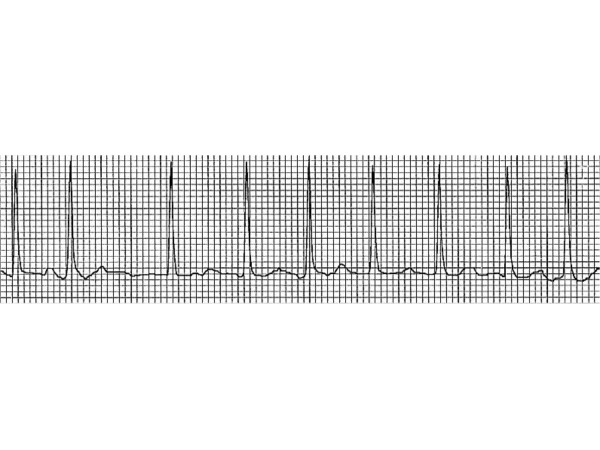
Electrocardiogram showing atrial fibrillation with rapid ventricular rate.

Four hours after presentation to ER, the admitting physician found the patient's heart rhythm to be irregular. A repeat EKG showed atrial fibrillation (Fig. 1) with a ventricular rate of 143/min. The patient was hemodynamically stable but was complaining of palpitations without dyspnea or chest pain. After 20 mg diltiazem had been administered intravenously, his ventricular rate dropped below 110/minute and the patient was started on metoprolol 25 mg twice daily orally. Troponin I level was below 0.04 ng/ml. Serum creatinine phosphokinase level was mildly elevated at 899 IU/liter with normal myoglobin (MB) fraction level. EKG performed after 12 hours revealed spontaneous reversion back to sinus rhythm.

**Figure 2:**
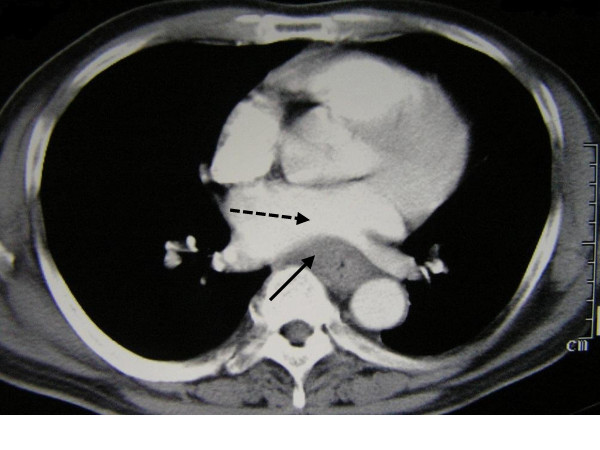
Computed tomography of the chest demonstrating esophageal thickening (compound line) compressing the left atrium (dashed line).

The next day, transthoracic echocardiography showed normal systolic and diastolic functions, and normal left atrium size without LVH. The patient underwent esophagogastroscopy which revealed a 10-cm, ulcerated mid-esophageal mass. The biopsy of the lesion showed infiltrating SCC. Computed tomography (CT) of the chest/abdomen/pelvis showed subcarinal lymphadenopathy and esophageal thickening compressing the left atrium (Fig. 2). Bronchoscopy revealed no abnormalities. Thyroid function tests, prostate specific antigen level, serum and urine protein electrophoreses were within normal limits.

During 15 days of hospitalization, no arrhythmia was witnessed again and he did not complain of palpitations, chest pain or dyspnea. The patient was discharged to have chemoradiotherapy as an outpatient.

## Discussion

To our knowledge, AF in association with EC was not reported previously except as a complication of esophagectomy and photodynamic therapy. In our case, direct mechanical compression of the left atrium by EC may have precipitated AF. On the other hand, AF might be related to the patient's HTN. In the Framingham study, the relative risk of AF in hypertensive patients with and without LVH was reported as 1.9 and 3.0, respectively [5]. Therefore, the risk of AF is modestly increased due to HTN in our patient who had a structurally normal heart on echocardiogram. Thus, we hypothesize that EC may have precipitated AF by compressing on the left atrium.

External compression of the left atrium was previously reported to precipitate AF. Upile *et al*. reported AF in a case with mega-esophagus due to achalasia in which AF was attributed to the external compression of the left atrium by food debris. AF had resolved after removal of food debris from the esophagus [6]. AF was also reported in a case with intrapericardial lipoma compressing the left atrium [7]. Similarly, swallowing may cause transient atrial tachyarrhythmias including AF, due to direct mechanical stimulation of the left atrium by the contents passing through the esophagus or activation of the autonomous nervous system [8]. It was demonstrated experimentally that in patients with swallowing induced tachyarrhythmias, inflation of a balloon in the esophagus at the level of the left atrium precipitated the tachyarrhythmias until the balloon was deflated [9]. In recent years, electrophysiological studies in patients with swallowing-induced tachyarrhythmias demonstrated ectopic atrial foci that were successfully treated with radiofrequency ablation [10]. Likewise, AF in our patient may have arisen from an automatic focus in the posterior left atrium which may be more excitable with mechanical stimulation. However, electrophysiological studies were not performed since AF was short-lived and did not recur.

The proximity of the esophagus to the left atrium may yield other unexpected complications. Atrial tachyarrhythmias may develop during esophagectomy and photodynamic therapy due to mechanical manipulation or thermal injury to the left atrium [3,4]. In reverse, atrioesophageal fistulas may develop during intraoperative or percutaneous catheter radioablation of pulmonary veins for treatment of atrial fibrillation [2]. The diminutive distance between the esophagus and left atrium may contribute to the occurrence of this complication. Additionally, Oishi *et al*. recently reported a case with syncope upon swallowing caused by an esophageal hiatal hernia compressing the left atrium and impeding the blood flow to the left ventricle [11].

## Conclusion

Esophageal cancer may precipitate AF by mechanical compression of the left atrium or pulmonary veins, triggering ectopic beats in susceptible patients. The proximity of the esophagus to the heart may be overlooked by physicians, but may have an important role in the pathogenesis of esophageal and heart disorders.

## Competing interests

The authors declare that they have no competing interests.

## Authors' contributions

UDB conceived of the report, treated the patient, gathered the data, searched the literature and drafted the manuscript. SB searched the literature and drafted the manuscript. AD treated the patient and conceived of the study. RRP and OIA treated the patient and helped to draft the manuscript. All authors read and approved the final manuscript.

## Consent

Written informed consent was obtained from the patient for publication of this case report and any accompanying images. A copy of the written consent is available for review by the Editor-in-Chief of this journal.
